# Molecular Alterations in Relation to Histopathological Characteristics in a Large Series of Pediatric Papillary Thyroid Carcinoma from a Single Institution

**DOI:** 10.3390/cancers13133123

**Published:** 2021-06-22

**Authors:** Elisabetta Macerola, Agnese Proietti, Anello Marcello Poma, Clara Ugolini, Liborio Torregrossa, Paola Vignali, Alessio Basolo, Gabriele Materazzi, Rossella Elisei, Ferruccio Santini, Fulvio Basolo

**Affiliations:** 1 Department of Surgical, Medical, Molecular Pathology and Critical Area, University of Pisa, 56126 Pisa, Italy; elisabetta.macerola@for.unipi.it (E.M.); a.proietti@ao-pisa.toscana.it (A.P.); marcellopoma@gmail.com (A.M.P.); clara.ugolini@unipi.it (C.U.); l.torregrossa@ao-pisa.toscana.it (L.T.); paola.vignali@phd.unipi.it (P.V.); gabriele.materazzi@med.unipi.it (G.M.); 2 Department of Clinical and Experimental Medicine, University of Pisa, 56126 Pisa, Italy; alessio.basolo@med.unipi.it (A.B.); rossella.elisei@med.unipi.it (R.E.); ferruccio.santini@med.unipi.it (F.S.)

**Keywords:** papillary thyroid carcinoma, pediatric thyroid cancer, molecular pathology, gene mutation, gene fusion

## Abstract

**Simple Summary:**

Papillary thyroid carcinoma (PTC) in pediatric patients shows different characteristics compared with PTC in adults. In this large mono-institutional cohort of pediatric PTCs, tumor tissue samples have been analyzed for point mutations and fusions. Overall, molecular alterations were detected in 131 out of 163 PTCs (80%). Fusion-driven PTCs showed more aggressive clinico-pathological features compared to mutation-driven and wild-type cases. These results highlight the importance of testing pediatric PTCs for gene rearrangement in routine characterization of tumors.

**Abstract:**

Papillary thyroid carcinoma (PTC) presents distinct clinico-pathological and molecular differences in children compared with adult patients. Whether the presence of rearrangements or point mutations is associated with aggressive PTC clinical presentation is still controversial. In this study, PTCs diagnosed in patients aged less than 18 years were retrospectively searched from the institutional archive and tumor tissue was tested for point mutations in *BRAF* and *RAS* genes and for rearrangements in *RET*, *NTRK1*, *NTRK3*, *ALK*, *PPARG*, *BRAF* and *THADA*. A total of 163 PTCs were analyzed. Point mutations were found in 83 (51%) and gene fusions in 48 cases (30%). The most frequent alteration was the *BRAF*^V600E^ mutation (36.8%), followed by *NTRK3* fusion (11%), *NRAS* mutation (10.4%) and *RET* fusion (10.4%). Fusion-driven PTCs showed more frequently infiltrative growth, larger tumors, extrathyroidal extension and N1b disease. PTCs showing solid growth pattern were significantly enriched in gene fusions. This is one of the largest cohorts of pediatric PTCs. Fusion-driven tumors most frequently show aggressive pathological features; the search for rearrangements, especially in tumors with solid areas, could improve the characterization of pediatric PTCs and offer possible therapeutic options.

## 1. Introduction

Papillary thyroid carcinoma (PTC) is the most common malignancy of the endocrine system. When occurring in pediatric age, PTC presents different clinico-pathological characteristics compared with PTC in adult patients [1]. In pediatric patients, PTC at presentation most frequently shows pathological features of aggressiveness, including lymph node metastases, extrathyroidal extension and distant metastases [2]. On the other hand, even in the presence of these characteristics that typically indicate poorer prognosis in adults, PTC pediatric patients generally have a favorable clinical outcome and an excellent survival [1,3,4]. The histology of PTC in children could also differ from that generally observed in adults: rare variants, such as the diffuse sclerosing variant, can represent a common finding in pediatric PTC cohorts [5]. 

According to the American Thyroid Association (ATA) guidelines for pediatric nodules and differentiated thyroid cancer (DTC), patients aged ≤18 years should be included in the pediatric age group. It is not clear whether further age subgroups (i.e., less than 10–15-year-old children) with different clinico-pathological characteristics could be identified [1].

From a molecular point of view, gene fusions acting as driver events in PTC tumorigenesis have shown to be more frequent in children than in adults, where the most frequent alterations involve point mutations in *BRAF* and *RAS* genes [1,6]. However, studies reveal a poor level of agreement on the reported frequencies of genetic alterations in pediatric thyroid tumors. For instance, over the last 10 years, *BRAF* mutations in pediatric PTC have been reported in frequencies ranging from 9 to 50% of patients [7,8,9,10,11,12]. *TERT* promoter mutations are associated with older age and, accordingly, their occurrence in pediatric PTCs is rare [12,13,14,15]. 

In a series of 27 PTCs in children, Prasad and colleagues found significant clinico-pathological differences between *BRAF*-driven and fusion-driven PTCs. In particular, fusion-positive PTCs more frequently showed larger size, infiltration of thyroid parenchyma, and lymphatic invasion [16]. If confirmed on a larger series, these results highlight the importance of analyzing not only classical point mutations, but also gene fusions in pediatric patients, even in the diagnostic setting. In the light of the recent approval of drugs specifically targeting rearranged genes, testing for gene fusions is emerging as an essential part of routine molecular pathology activity.

In this retrospective cohort study, a consecutive series of PTC patients aged less than 18 years was tested for the presence of molecular alterations, including the most frequent point mutations and rearrangements. Clinico-pathological features of PTC were correlated to the type of molecular alteration.

## 2. Materials and Methods

A search for cases diagnosed as PTC in pediatric patients was conducted retrospectively and anonymously in the archives of the Anatomical Pathology Section of the University Hospital of Pisa for the time interval 2014–2020. Inclusion criteria were the following: age ≤18 years; histological diagnosis of PTC; availability of tumor tissue for molecular analysis. All samples were carefully reviewed by three pathologists with experience in thyroid pathology [17]; as well as the PTC variant, growth patterns, such as solid, tall cell, hobnail were recorded whenever present in more than 5% of the tumor. The following clinico-pathological characteristics of PTCs were recorded: tumor size, degree of local invasiveness (presence of complete encapsulation, encapsulation with invasion of tumor capsule or infiltration of thyroid parenchyma), presence of extrathyroidal extension, lymphovascular invasion, multifocality and American Joint Committee on Cancer T, N and M stages. Noninvasive follicular neoplasm with papillary-like nuclear features (NIFTP) diagnosed since 2016 were also considered in this study. 

DNA and RNA were purified from formalin-fixed, paraffin-embedded tissue blocks by the Qiamp DNA Mini kit and the RNeasy FFPE kit, respectively (Qiagen, Hilden, Germany). The most suitable paraffin block was selected for each case, and 2–4 10 µm-thick sections were obtained. After standard deparaffinization and rehydration of sections, tumor areas were collected by manual macrodissection and placed in a tube containing the lysis buffer. All the subsequent downstream procedures for nucleic acid purification were conducted according to the manufacturer’s instructions. 

DNA samples were analyzed for the presence of mutations in *BRAF* exon 15 and *NRAS*, *HRAS* and *KRAS* exon 3 by using real-time PCR and high-resolution melt analysis (Type-it HRM PCR Kit, Qiagen). PCR products of samples with altered melt curves were purified and analyzed by direct sequencing on an AbiPrism 3130 Genetic Analyzer (Applied Biosystem, Foster City, CA, USA).

For rearrangement analysis, an nCounter custom panel was designed, able to detect the main fusion events found in thyroid cancer (Nanostring Technologies, Seattle, WA, USA). The codeset included eight probes targeting the 3’-end and the 5’-end of six genes to detect expression imbalance: *ALK*, *BRAF*, *NTRK1*, *NTRK3*, *RET*, *THADA*; this strategy was not applicable to *PPARG*, since the typical breakpoint occurs at the very 5’-end of the gene. Moreover, 29 breakpoint-specific probes targeting the most frequent fusion partners, such as *CCDC6/RET*, *PAX8/PPARG*, *ETV6/NTRK3*, *EML4/ALK* were included. nCounter chemistry is designed to allow hybridization of the reporter probe, which carries the color-coded signal, at the 3’-end of transcripts. Therefore, all the breakpoint-specific probes targeting the same 3’-end partners share the same signal and the partner cannot be unambiguously distinguished.

Considering that gene fusions and mutations should be mutually exclusive, only point mutation-negative samples were tested by the nCounter for the presence of rearrangements, along with a subgroup of mutation-positive samples as negative controls. We also analyzed four clinical samples positive for known rearrangements whose RNA was already available (all from thyroid tumors, two with *RET/PTC1*, one with *PAX8/PPARG* fusion, one with *TPM3/NTRK1* fusion). The nCounter analysis was performed according to the manufacturer’s instructions using 400 ng of RNA per sample. Raw count data obtained by the nCounter Digital Analyzer were exported and analyzed using the nSolver analysis software v4.0 (Nanostring Technologies). All samples were first submitted to the software quality check; raw data were then analyzed with the Advanced analysis module v2.0 (Nanostring Technologies) following the manufacturer’s instructions. The positivity was based on a significant imbalance between 5’-end and 3’-end probes and/or a signal above the negative control threshold of the breakpoint-specific probes. All suspected fusion events were confirmed by a second test. In detail, the *RET*, *PPARG* and *NTRK* positive samples were tested by retro-transcription real-time PCR (RT-PCR), which contains specific probes for the most frequent fusion events (EasyPGX ready thyroid fusion and EasyPGX ready NTRK fusion, Diatech Pharmacogenetics, Jesi, AN, Italy). Since RT-PCR assays contain a limited number of probes identifying only the most frequent fusion events, FISH testing was performed on tissue slides in case of PCR-negative results, to assess the presence of rearrangements involving rare partners. For *ALK* positive samples, RT-PCR assay was not available, and FISH was used as a second confirmation technique. *ALK* (Vysis ALK Break Apart FISH Probe Kit, Abbott Laboratories, Chicago, IL, USA), *RET* (Vysis 10q11 RET Break-Apart FISH Probe Kit, Abbott Laboratories), *NTRK1* (ZytoLight SPEC NTRK1 Dual Color Break Apart Probe, Zytovision GmbH, Bremerhaven, Germany) and *NTRK3* (ZytoLight SPEC NTRK3 Dual Color Break Apart Probe, Zytovision GmbH) FISH analyses have been performed on unstained tissue slides according to manufacturer’s protocols. Confirmation by next-generation sequencing (NGS) analysis would have been preferred, but FISH choice was necessary because of the scarce quality of RNA obtained from archival paraffined samples in terms of the high level of fragmentation.

Molecular alteration data were formatted as mutation annotation file (MAF) and the oncoplot was built using the procedures of the maftools package (v.3.12). The alluvial plot showing the relationship between gene fusions, tumor histotypes and additional growth patterns was plotted by using the ggalluvial package (v.0.12.3). Statistical analysis was conducted on molecular data to identify differences in terms of clinico-pathological tumor features; moreover, the cases were divided into two classes according to the age of the patients, <15-year-olds and ≥15-year-olds. The categorical data were analyzed by the Pearson Chi-squared test or the Fisher exact test whenever appropriate. The Mann–Whitney test or the Kruskal–Wallis test and the Dunn test for pairwise comparisons were used for continuous variables. *p*-values below 0.05 were considered significant. All analyses were performed in the R environment (https:/www.R-project.com, v.4.0.2, last accessed on 11 May 2021).

## 3. Results

### 3.1. Cohort Characteristics

A total of 187 records were retrieved. Fifteen cases were discarded because the diagnosis of PTC (size range 0.2–0.4 cm) was an incidental finding within the context of multinodular goiters. Nine cases were excluded because of insufficient (*n* = 4) or unavailable tumor tissue (*n* = 5) for molecular analysis. 

A series of 163 samples was then analyzed for genetic mutations and rearrangements. The clinico-pathological characteristics of tumors are summarized in Table 1. Data on the presence of distant metastases at diagnosis were available in 70 patients; two patients (2.9%) presented with distant metastases. Median age was 16 years (range 8–18). The cohort included 116 females and 47 males, with female/male ratio of 2.5. The diagnosis was classical PTC in 99 cases (C-PTC, 60.7%), follicular variant in 34 (FV-PTC, 20.9%), solid variant in nine (SV-PTC, 5.5%), tall cell variant in seven (TCV-PTC, 4.3%), diffuse sclerosing variant in four (DSV-PTC, 2.5%) and the Hürthle cell variant in one case (HCV-PTC 0.6%). Moreover, there were eight NIFTPs (4.9%) and one rare case of PTC lymph node metastasis with no identifiable primary tumor within the thyroid gland (0.6%). Besides the PTC variants, the following growth patterns were identified: among the 99 C-PTCs, tall cell areas were present in 27 (27.3%), solid/trabecular areas in 22 (22.2%), follicular areas in four (4%), columnar cell areas in three (3%), mixed micropapillary/hobnail areas in one (1%), and mixed hobnail/clear cells areas in one (1%). Solid/trabecular areas were also present in 13 out of the 34 FV-PTCs (38.2%) and in one out of the seven TCV-PTCs (14.3%).

### 3.2. Molecular Analysis

**Figure 1:**
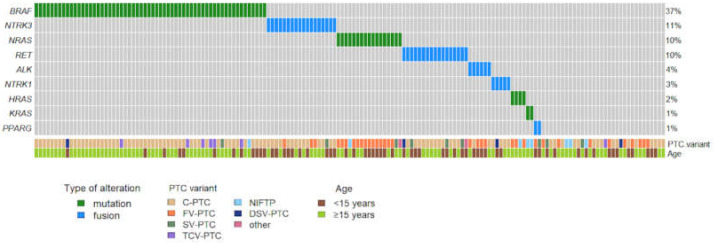
Oncoplot showing the prevalence and type of molecular alterations detected in 163 pediatric PTCs. Sidebars show the PTC variant and the age class of patients. Abbreviations: C-PTC, classical papillary thyroid carcinoma; FV-PTC, follicular variant papillary thyroid carcinoma; SV-PTC, solid variant papillary thyroid carcinoma; TCV-PTC, tall cell variant papillary thyroid carcinoma; DSV-PTC, diffuse sclerosing variant papillary thyroid carcinoma; NIFTP, noninvasive follicular neoplasm with papillary-like nuclear features.

Figure 1 shows details of the distribution of molecular alterations according to PTC variants and age class. Overall, point mutations in *BRAF* and *RAS* genes were present in 83 out of 163 PTCs (51% of all samples). In detail, 59 out of 163 PTCs had the *BRAF*^V600E^ mutation (36.2%); one case was mutated in *BRAF* codon 599 (p. T599I). Mutations in the *RAS* genes were detected in 23 PTCs (14%) and were distributed as follows: *NRAS* codon 61 mutations in 17 (10%), *HRAS* codon 61 mutations in four (2.5%), *KRAS* codon 61 mutations in two samples (1.2%). *BRAF* mutations were found almost exclusively in C-PTCs (*n* = 51) and TCV-PTCs (*n* = 6); moreover, *BRAF* mutation was present in one DSV-PTC, one SV-PTC, and one NIFTP, the latter harboring the *BRAF*^T599I^. *RAS* mutations were found in 17 FV-PTCs, four NIFTPs, one SV-PTC and one HCV-PTC.

nCounter analysis was performed in all the 80 mutation-negative PTCs, in a subset of eight *BRAF*- or *RAS*-positive samples and four fusion-positive clinical samples. No fusion events were detected in the eight point-mutation-positive samples, and all the clinical samples were correctly confirmed.

**Figure 2:**
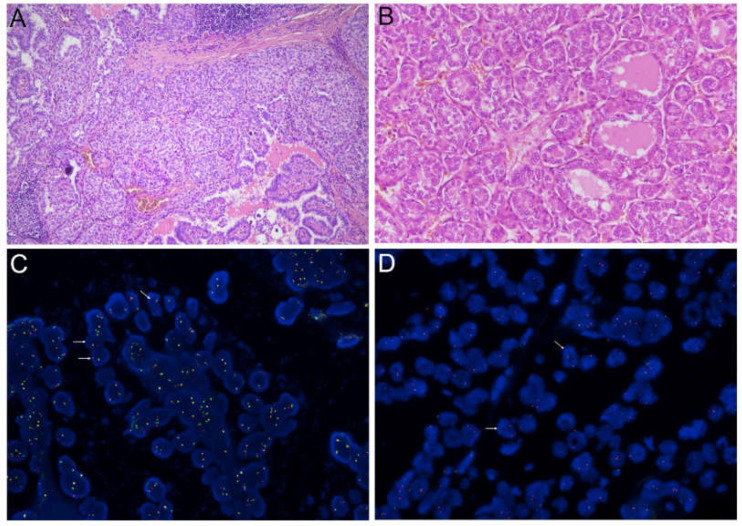
Microscopic images showing histological details and FISH analysis of two PTCs. (**A**) C-PTC with solid areas positive for *NTRK3* fusion (hematoxylin and eosin staining, original magnification 10×); (**B**) detail of follicular growth areas of an *ALK*-positive C-PTC (hematoxylin and eosin staining, original magnification 20×); corresponding FISH analyses of *NTRK3* and *ALK* are shown in (**C**) and (**D**), respectively. White arrows indicate examples of positive nuclei.

Gene fusions were detected in 48 PTCs. *NTRK3*, the most frequent rearranged gene, was found in 18 samples, followed by *RET* in 17 samples, *ALK* in 6 samples, *NTRK1* in 5 samples, and *PPARG* in 2 samples. No fusions involving the *BRAF* and *THADA* genes were identified. The nCounter analysis displayed an important technical limitation for *NTRK3* fusion detection. In detail, the majority of cases presented a basal overexpression of the 5’-end transcripts of the *NTRK3* gene (median 3’/5’ ratio in all samples: 0.2). Consequently, fusion positive samples were identified by the analysis software only when they showed *ETV6/NTRK3* breakpoint-specific counts, while no sample was positive for 3’/5’ imbalance. All the *ETV6/NTRK3* positive samples (*n* = 12) were confirmed as rearranged by RT-PCR; all samples showing 3’-end mean counts above the negative count threshold were analyzed by FISH, which was positive in six additional cases. The *RET* fusion partner was identified by RT-PCR in 15 out of 17 cases: 14 presented the *CCDC6/RET* fusion (*RET/PTC1*) and one the *NCOA4/RET* fusion (*RET/PTC3*). The six *ALK*-positive samples were all confirmed by FISH. With regard to *NTRK1*, two samples were confirmed by RT-PCR, while the remaining three were confirmed by FISH. Two examples of PTC histological images and corresponding FISH analyses are shown in Figure 2.

According to the PTC variants, *RET* and *NTRK* fusions were detected mainly in C-PTCs (12 C-PTCs out of 17 *RET* positive cases; 18 C-PTCs out of 23 *NTRK* positive cases), while *ALK* was more prevalent in FV-PTC (four FV-PTCs out of six *ALK* positive cases). Five out of eight NIFTPs (62.5%) had point mutations, and none was positive for gene fusions. 

Statistical differences in fusion-driven PTCs compared with mutated-PTCs and wild-type PTCs were observed in terms of minor patient age, greater tumor size, lower rate of tumor encapsulation and higher rates of extra-thyroidal extension and N1b lymph nodes metastases (Table 2). 

**Figure 3:**
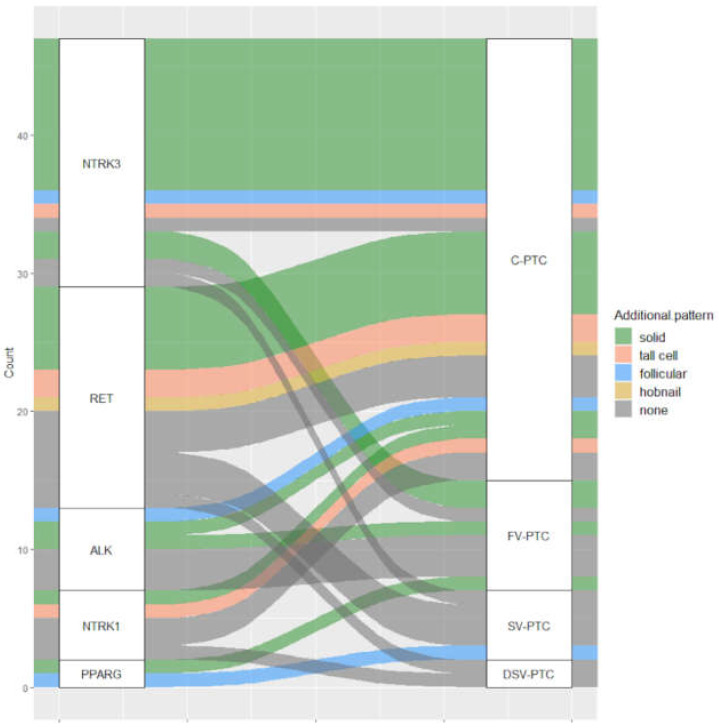
Alluvial plot representing fusion-positive PTC. Correspondence between rearranged gene, PTC variant and presence of histological growth patterns (additional pattern) is shown. The case of PTC lymph node metastasis with no primary tumor has been excluded from this plot. Abbreviations: C-PTC, classical papillary thyroid carcinoma; FV-PTC, follicular variant papillary thyroid carcinoma; SV-PTC, solid variant papillary thyroid carcinoma; DSV-PTC, diffuse sclerosing variant papillary thyroid carcinoma.

PTCs of patients aged less than 15 years were more frequently rearranged (*p*-value 0.0018). As concerns the secondary growth pattern areas, gene fusions were significantly more prevalent in PTCs with a solid component (*p*-value < 0.0001), since they were positive in 23 out of 36 cases (64%). The distribution of gene fusion according to histotype and secondary growth pattern areas is shown in Figure 3.

The presence of histo-pathological differences between patients aged < 15 years (*n* = 61) and those aged ≥ 15 years (*n* = 102) was also investigated. Among all the above considered parameters, only tumor infiltration (*p*-value = 0.0031) and PTC variant distribution (*p*-value = 0.0361) were significantly different according to the age class: tumors were generally infiltrative in patients younger than 15 years and C-PTCs were less represented in this age group, while FV-PTCs and SV-PTCs occurred more frequently in younger patients.

## 4. Discussion

Pediatric PTCs represent a distinct clinico-pathological entity compared with adult PTCs. From a molecular point of view, the literature data indicate that PTCs in pediatric patients show a higher prevalence of gene rearrangements than PTCs in adult patients, while there are conflicting results about the frequency of point mutations. We collected one of the largest series of pediatric PTCs, including 163 tumors from patients aged ≤18 years, in order to investigate the prevalence of the most frequent gene mutations and fusions and to describe how these molecular alterations are distributed according to PTC variants and clinico-pathological tumor features. Overall, 131 PTCs out of 163 (80%) were positive for a molecular alteration; in particular, point mutations were found in 83 (51%) and gene fusions in 48 cases (30%). The *BRAF* mutation rate observed in the present cohort (37%) is higher than that observed in the majority of studies on pediatric PTCs [7,9,12,14,15,16], but even higher frequencies have been reported [8,11]. The prevalence of BRAF mutations, mostly detected in C-PTCs and TCV-PTCs, is directly related to the relative abundance of these variants in the study cohort. In this regard, previous studies on pediatric PTCs have reported that C-PTC may constitute 28–71% of all cases, which is in line with their prevalence in our cohort (60%) [7,9,12,14,15,16]. The presence of tall cell areas in C-PTCs is a normal finding in adult patients. In our series, 28.3% of pediatric PTCs presented a tall cell component. To our knowledge, no detailed reports have been conducted on this specific aspect in pediatric PTCs; therefore, we cannot make comparisons to assess the impact of our findings. However, it should be noted that the relative abundance of TCV-PTC in our cohort (4%) is actually higher than has been previously reported [7,9,12,14,15,16]. This might be partly due to the changes in the criteria for TCV-PTC histological diagnosis. These changes, introduced in 2017, are likely to cause an overall increase of its occurrence [17]. 

In molecular pathology laboratories, the analysis of rearrangements directly on mRNA purified from tumor tissue has some limitations. In particular, RT-PCR is a highly sensitive method, but it requires large amounts of RNA and can detect only small sets of known fusion events; on the other hand, the analysis by NGS requires smaller RNA amounts, but it is greatly affected by the scarce quality of RNA obtained from paraffined specimens [18]. The nCounter system could bridge the gap between these two techniques, since it does not require large amounts of RNA as RT-PCR and it tolerates nucleic acid degradation better than NGS. Indeed, the nCounter platform is based on the direct hybridization of specific color-barcoded probes with the target RNA molecules in a sample. Transcript quantity is determined by digital counting of the hybridized complexes. Therefore, samples are not subject to retro-transcription nor to amplification processes. In our series, all the analyzed samples passed the nCounter software quality check.

*NTRK3* was the most frequent rearranged gene, occurring in 18 PTCs (11%), with *ETV6* being the most common 5’-end partner (in 12 out of 18 cases). A similar frequency has been previously reported by several authors [7,12,14], and in one study *NTRK3* fusion was detected in 22% of DTC (16). 

Fusion-positive PTC showed significantly higher rates of aggressive features, including greater tumor size, infiltration of thyroid parenchyma, extra-thyroidal extension, lymphovascular invasion, and N1b lymph node metastases. Similar associations were reported by Pekova and colleagues in a series of 93 PTC patients aged <20 years [12]. Interestingly, the authors found that *NTRK3* was significantly more prevalent in FV-PTC, but this observation was not confirmed in our cohort. On the basis of current literature data, *NTRK*-positive PTC frequently show a mixed papillary/follicular growth pattern or even predominantly follicular growth [19,20], which in our cohort was observed in only one out of 18 cases (Figure 3). A direct comparison with the cancer genome atlas (TCGA) adult series cannot be made, but it is worth noting that *NTRK3* rearranged PTCs, as well as *ALK* and *NTRK1*, were not consistently and homogeneously defined as *BRAF*-like or *RAS*-like tumors [6]. 

In our series, the distribution of fusion events among PTC variants was not significantly different form mutation-driven and wild-type PTCs. However, TCV-PTC and NIFTP did not harbor any rearrangement, while five out of nine SV-PTCs (55%) were fusion-positive. Furthermore, 36 out of 163 PTCs (22.1%) showed areas of solid/trabecular growth; these findings are not unusual in pediatric PTCs [17]. Samuels reported a series of 18 pediatric FV-PTCs and four tumors (22%) presented a mixed follicular-solid/trabecular pattern [21]. Interestingly, in our PTC series, tumors with solid areas were significantly enriched in gene rearrangements. While SV-PTCs are known to frequently harbor *RET/PTC* fusions, on the basis of literature data, the relatively high prevalence of *NTRK* fusions in tumors with a solid component was partially unexpected. Indeed, only one study reported that six tumors out of seven *NTRK* rearranged pediatric PTCs showed at least a minor solid component, in line with our results [16]. These peculiar histo-pathological and molecular aspects involving PTCs of any variant with solid growth areas deserve further investigation.

In this study, gene fusions involving *BRAF* and *THADA* were not detected. There are few studies investigating *BRAF* fusion in pediatric thyroid cancer patients. For instance, the *AGK/BRAF* fusion was specifically investigated in 80 pediatric PTCs by RT-PCR, and was detected in 15 (19%) [10]; another study found *AGK/BRAF* in 4 out of 35 (11%) pediatric PTCs (7); Pekova and collaborators failed to identify *AGK/BRAF* events, but found BRAF rearrangements in 2 out of 93 (4%) cases (12). Even less data are available for *THADA* fusions, quite frequent in adult *RAS*-like thyroid tumors [6,22], but whose incidence in pediatric PTCs is still unclear.

Some authors raised the possibility that, according to clinico-pathological tumor features such as local invasiveness and distant metastases at presentation, pediatric PTCs could be further divided in a subclass of patients aged 14 years or less [1]. In our cohort, gene fusion-positive PTC patients were significantly younger (median age 14 years) than those affected by fusion-negative tumors, and fusion-positive cases showed higher rates of aggressive features. On the other hand, the analysis of the subgroup of patients aged less than 15 years revealed that the infiltrative pattern was the only significantly different parameter in terms of tumor aggressiveness. These findings indicate that the presence of rearrangements and not younger age might explain a greater risk of extensive disease in younger patients occasionally observed by some authors and not confirmed by others [1,23,24,25,26]. 

Although pediatric DTCs show excellent survival, clinical guidelines highlight the importance of a correct risk stratification of patients, to establish the probability of persistent/recurrent disease and to define appropriate therapeutic strategies. The prognostic impact of several clinico-pathological factors, such as tumor size, histotype and local invasion in pediatric DTC patients remains controversial [1,2]. 

An important limitation of this study is the lack of clinical follow-up data. This would have allowed to also investigate the real impact of different molecular and pathological PTC characteristics in the prognostic risk stratification of patients.

## 5. Conclusions

Our results show that gene fusions, mainly present in younger patients and in solid growth pattern PTCs, correlate with aggressive pathological tumor features. Furthermore, the presence of rearrangements is associated with the N1b disease, which represents a paramount criterium for patient risk stratification according to the ATA guidelines for pediatric DTC [1]. It is still not clear whether the preoperative molecular testing of pediatric thyroid nodules could help define the extension of surgery, thus reducing the rate of repeat surgery; in the same way, to what extent somatic alterations detected in pediatric PTCs could serve as prognostic markers has yet to be fully addressed. Nevertheless, our findings confirm the importance of extending the molecular screening of pediatric PTCs to gene fusion testing.

## Figures and Tables

**Table 1:** Cohort description.

Pediatric PTCs *n* = 163		
Age		
	median, range	16, 8–18
	<15-year-old	61 (37.4%)
	≥15-year-old	102 (62.6%)
Sex		
	Female	116 (71.2%)
	Male	47 (28.8)
Tumor size [cm]		
	mean (SD)	2.3 (1.3)
PTC variant		
	C-PTC	99 (60.7%)
	FV-PTC	34 (20.9%)
	SV-PTC	9 (5.5%)
	TCV-PTC	7 (4.3%)
	DSV-PTC	4 (2.5%)
	HCV-PTC	1 (0.6%)
Metastasis without primary tumor *		1 (0.6%)
	NIFTP *	8 (4.9%)
Tumor invasiveness		
Totally encapsulated		14 (9.1%)
Encapsulated with tumor capsule invasion		20 (13.0%)
Infiltration of thyroid parenchyma		120 (77.9%)
Extrathyroidal extension		
	Minimal	47 (30.5%)
	Gross	1 (0.7%)
	Absent	106 (68.8%)
Multifocality		
	Present	69 (44.8%)
	Absent	85 (55.2%)
Lymphovascular invasion		
	Present	65 (42.2%)
	Absent	89 (57.8%)
Tumor stage		
	T1a	20 (13.0%)
	T1b	68 (44.1%)
	T2	45 (29.2%)
	T3a	20 (13.0%)
	T4a	1 (0.7%)
Lymph node metastasis		
	N0	22 (14.3%)
	N1a	35 (22.7%)
	N1b	27 (17.5%)
	NA	70 (45.5%)

Abbreviations: PTC, papillary thyroid carcinoma; C-PTC, classical papillary thyroid carcinoma; FV-PTC, follicular variant papillary thyroid carcinoma; SV-PTC, solid variant papillary thyroid carcinoma; TCV-PTC, tall cell variant papillary thyroid carcinoma; DSV-PTC, diffuse sclerosing variant papillary thyroid carcinoma; HCV-PTC, Hürthle cell variant papillary thyroid carcinoma; NIFTP, noninvasive follicular neoplasm with papillary-like nuclear features; NA, not available. * Excluded from further clinical-pathological characterization.

**Table 2:** Statistical comparison between fusion-driven and point mutation-driven pediatric PTCs.

		Fusion *n* = 48	Mutation *n* = 83	Wild-Type *n* = 32	*p*-Value
*Age*					
	median, range	14, 8–18	17, 8–18	16, 9–18	**< 0.0001**
	<15-year-old	27 (56.3%)	21 (25.3%)	13 (40.6%)	**0.0018**
	≥15-year-old	21 (43.7%)	62 (74.7%)	19 (59.3%)	
*Sex*					
	Female	34 (70.8%)	59 (71.1%)	23 (71.9%)	0.9947
	Male	14 (29.2%)	24 (28.9%)	9 (28.1%)	
*Tumor size [cm]*					
	mean (SD)	2.6 (1.2)	2.1 (1.3)	2.3 (1.5)	**0.0200**
*PTC variant*					
	C-PTC	32 (66.7%)	51 (61.4%)	16 (50.0%)	0.0955
	FV-PTC	8 (16.7%)	17 (20.5%)	9 (28.1%)	
	SV-PTC	5 (10.4%)	2 (2.4%)	2 (6.2%)	
	TCV-PTC	0	6 (7.2%)	1 (3.1%)	
	DSV-PTC	2 (4.2%)	1 (1.2%)	1 (3.1%)	
	Other ^a^	1 (2.1%)	1 (1.2%)	0	
	NIFTP ^b^	0	5 (6.0%)	3 (9.4%)	
*Tumor invasiveness*					
Totally encapsulated		0	6 (7.7%)	8 (27.6%)	**0.0003**
Encapsulated with tumor capsule invasion		3 (6.4%)	10 (12.8%)	7 (24.1%)	
Infiltration of thyroid parenchyma		44 (93.6%)	62 (79.5%)	14 (48.3%)	
*Extrathyroidal extension*					
Present ^c^		24 (51.1%)	21 (26.9%)	3 (10.3%)	**0.0005**
	Absent	23 (48.9%)	57 (73.1%)	26 (89.7%)	
*Multifocality*					
	Present	27 (57.4%)	33 (42.3%)	9 (31.0%)	0.0652
	Absent	20 (42.6%)	45 (57.7%)	20 (69.0%)	
*Lymphovascular invasion*					
Present		31 (66.0%)	29 (37.2%)	5 (17.2%)	**< 0.0001**
	Absent	16 (34.0%)	49 (62.8%)	24 (82.8%)	
*Tumor stage ^d^*					
	T1a	2 (4.2%)	14 (18.0%)	4 (13.8%)	0.0783
	T1b	17 (36.2%)	37 (47.4%)	14 (48.3%)	
	T2	20 (42.6%)	17 (21.8%)	8 (27.6%)	
	T3a	7 (14.9%)	10 (12.8%)	3 (10.3%)	
	T4a	1 (2.1%)	0	0	
*Lymph node metastasis*					
	N0	7 (14.9%)	10 (12.8%)	5 (17.2%)	**0.0489**
	N1a	11 (23.4%)	18 (23.1%)	6 (20.7%)	
	N1b	18 (38.3%)	6 (7.7%)	3 (10.3%)	
	NA	11 (23.4%)	44 (56.4%)	15 (51.7%)	

Statistically significant comparisons are reported in bold in the “*p*-value”column. Abbreviations: SD, standard deviation; PTC, papillary thyroid carcinoma; C-PTC, classical papillary thyroid carcinoma; FV-PTC, follicular variant papillary thyroid carcinoma; SV-PTC, solid variant papillary thyroid carcinoma; TCV-PTC, tall cell variant papillary thyroid carcinoma; DSV-PTC, diffuse sclerosing variant papillary thyroid carcinoma; NIFTP, noninvasive follicular neoplasm with papillary-like nuclear features; NA, not available. ^a^ Includes one Hürthle cell PTC and one PTC metastasis without primary tumor. ^b^ Excluded from statistical comparisons involving clinico-pathological features. ^c^ Minimal and gross extrathyroidal extension considered together for statistical analysis. ^d^ T1a plus T1b and T3a plus T4a considered as unique categories for statistical analysis.

## Data Availability

No new data were created or analyzed in this study. Data sharing is not applicable to this article.
